# Prevalence and Predictors of Maternal Anemia during Pregnancy in Gondar, Northwest Ethiopia: An Institutional Based Cross-Sectional Study

**DOI:** 10.1155/2014/108593

**Published:** 2014-01-20

**Authors:** Mulugeta Melku, Zelalem Addis, Meseret Alem, Bamlaku Enawgaw

**Affiliations:** ^1^Department of Hematology, School of Biomedical and Laboratory Sciences, College of Medicine and Health Sciences, University of Gondar, P.O. Box 196, 6200 Gondar, Ethiopia; ^2^Department of Medical Microbiology, School of Biomedical and Laboratory Sciences, College of Medicine and Health Sciences, University of Gondar, 6200 Gondar, Ethiopia; ^3^Department of Immunology and Molecular Biology, School of Biomedical and Laboratory Sciences, College of Medicine and Health Sciences, University of Gondar, 6200 Gondar, Ethiopia; ^4^Department of Hematology, School of Biomedical and Laboratory Sciences, College of Medicine and Health Sciences, University of Gondar, 6200 Gondar, Ethiopia

## Abstract

*Background*. Anaemia is a global public health problem which has an eminence impact on pregnant mother. The aim of this study was to assess the prevalence and predictors of maternal anemia. *Method*. A cross-sectional study was conducted from March 1 to April 30, 2012, on 302 pregnant women who attended antenatal care at Gondar University Hospital. Interview-based questionnaire, clinical history, and laboratory tests were used to obtain data. Bivariate and multivariate logistic regression was used to identify predictors. *Result*. The prevalence of anemia was 16.6%. Majority were mild type (64%) and morphologically normocytic normochromic (76%) anemia. Anemia was high at third trimester (18.9%). Low family income (AOR [95% CI] = 3.1 [1.19, 8.33]), large family size (AOR [95% CI] = 4.14 [4.13, 10.52]), *hookworm* infection (AOR [95% CI] = 2.72 [1.04, 7.25]), and *HIV *infection (AOR [95% CI] = 5.75 [2.40, 13.69]) were independent predictors of anemia. *Conclusion*. The prevalence of anemia was high; mild type and normocytic normochromic anemia was dominant. Low income, large family size, *hookworm* infection, and HIV infection were associated with anemia. Hence, efforts should be made for early diagnosis and management of *HIV* and *hookworm* infection with special emphasis on those having low income and large family size.

## 1. Background

Anaemia is a global public health problem affecting both developing and developed countries with major consequences for human health as well as social and economic development which results in a loss of billions of dollars annually [1–3]. According to the 2008 World Health Organization (WHO) report, anaemia affected 1.62 billion (24.8%) people globally [2]. It had an estimated global prevalence of 42% in pregnant women and is a major cause of maternal mortality [4, 5]. In Africa, 57.1% of the pregnant women were anemic. Moreover, anemia in pregnant women is a severe public health problem in Ethiopia; 62.7% of pregnant women were anemic [2]. Although the prevalence varies widely in different settings and accurate data are often lacking, in resource-limited areas terribly significant proportions of women of childbearing age particularly pregnant are anaemic [3]. Geographically, those living in Asia and Africa are at the greatest risk [1].

The effect of anemia during pregnancy on maternal and neonatal life ranges from varying degrees of morbidity to mortality. As many studies elucidated, severe anemia (Hg < 7 g/L) during pregnancy has been associated with major maternal and fetal complications. It increases the risk of preterm delivery [6, 7], low birth weight [6–9], intrauterine fetal death [7], neonatal death [10], maternal mortality [11], and infant mortality [12].

Anemia is multifactorial in etiology; the disease is thought to be mainly caused by iron deficiency in developing countries. In sub-Saharan Africa where iron deficiency is common, the prevalence of anemia has often been used as a proxy for iron deficiency anemia (IDA) [3]. Other micronutrient deficiency (vitamins A and B12, riboflavin, and folic acid) has also been a cause of anemia during pregnancy [13]. Likewise, Infectious diseases such as malaria, helminthes infestations, and HIV are also implicated with high prevalence of anemia in sub-Saharan Africa [14, 15]. There was also a considerable variation in the prevalence of pregnancy anemia because of the differences in socioeconomic conditions, lifestyles, and health seeking behaviors of different population across different countries and cultures and obstetrics and gynecological related condition of pregnant mothers [16–41].

Since anaemia during pregnancy has a deleterious consequences, WHO adopted reducing maternal mortality as one of the three health-related millennium development goals so that international community is committing within this framework to reduce maternal mortality by three quarter at the end of 2015 [42]. Anemia prevalence data remains an important indicator of public health since anemia is related to morbidity and mortality in the population groups usually considered to be the most vulnerable like pregnant women. At a global level, anemia prevalence is a useful indicator to assess the impact of widespread or highly effective interventions and to track the progress made towards the goal of reducing anemia during pregnancy [43]. Anemia prevalence study is also useful to monitor the progress of reproductive health [2]. Despite the efforts made to reduce the burden, its prevalence has not been studied yet comprehensively in developing countries. Thus, the objective of this study was to determine the prevalence and predictors of anemia among pregnant women who attended ANC in Gondar University Hospital.

## 2. Methods

### 2.1. Study Population, Sample Size, and Sampling Procedure

The study population was pregnant mothers attending antenatal care (ANC) at Gondar University Teaching Hospital. The hospital is found in Gondar town under Amhara regional state of Ethiopia which is located at 750 Km far from Addis Ababa, the capital city of Ethiopia, to the Northwest part of the country. The town is situated at an altitude of 2100 to 2870 meters above the sea level. According to the 2007 Ethiopian census report, Gondar has a total population of 206 and 987 and more than half (108, 902) of them are females [44].

A single population proportion formula, [*n* = (*Zα*/2)^2^ *p*(1 − *p*)/*d* ^2^], was used to estimate the sample size. However, due to the lack of previous studies about the prevalence of anemia during pregnancy in this particular area, 50% prevalence was used for calculation. By reviewing the records of daily flow of pregnant women for ANC utilization, about 1410 pregnant women were estimated to visit ANC clinic during the study period. Since the population during the study period was below 10,000, the sample correction formula was applied. Then, a total of 302 pregnant women who attended ANC service were selected using systematic random sampling technique from their sequence of ANC visit in the period between March and April, 2012, for two months.

### 2.2. Data Collection

A face-to-face interview using structured pretested questionnaire was employed to obtain data about sociodemographic, obstetric, and gynecological, dietary intake, and medical conditions of pregnant mothers. As for the current pregnancy, intake of haematinics, gestational age, ante partum hemorrhage, and dietary intake were documented. Blood pressure, weight, and height were measured and body mass index (BMI) was calculated as (weight (kg)/height (m^2^)). Women were then categorized into four groups according to their BMI as follows: underweight (BMI ≤ 20 kg/m^2^), normal (20 kg/m^2^ ≤ BMI ≤ 24.9 kg/m^2^), overweight (BMI of 25 kg/m^2^ ≤ BMI ≤ 29.9 kg/m^2^), and obese (BMI ≥ 30 kg/m^2^) [23]. A total of 6 mL venous blood sample was obtained from each participant. Of this, 3 mL of it was drawn into ethylene diamine tetraacetic acid tube for complete blood count whereas the remaining 3 mL was drawn to plane tube for serological tests. Participants were also requested to give fresh stool sample for parasitological examination of intestinal parasitosis.

### 2.3. Laboratory Analysis

Complete blood count including red blood cell count, hemoglobin concentration (Hgb), mean cell volume (MCV), mean cell hemoglobin (MCH), and mean cell hemoglobin concentration (MCHC), platelet count, and white blood cell count were carried out using SYXMEX KX-21 haematology analyzer (Sysmex Corporation Kobe, Japan). A thin and thick blood film had been prepared and stained with Giemsa stain for the detection and speciation of *Plasmodium* parasite species. Stool wet mount was prepared using saline and/or iodine and examined microscopically for identification of intestinal helminthes and protozoa parasitosis. All stool samples were processed within 30 minutes of collection. Serum and/or plasma samples were tested for HIV following the current HIV1/2 testing algorism using KHB (Shanghai Kehua bio-engineering Co., LTD., China), Stat-pack (Chembio Diagnostic Systems, Inc., New york, USA), and Uni-gold (Trinity Biotech Plc, Bray, Ireland). Syphilis reactivity was also tested using RPR test (Human GmbH-Wiesbaden, Germany) as per the manufacturer's instruction and recommendation.

### 2.4. Assessment of Anemia

Hgb cutoff value adjusted to sea level altitude was used to define anemia on the basis of gestational age and to classify the degree of severity using WHO criteria. The Hgb value less than 11.0 g/dL at first and third trimesters and less than 10.5 g/dL at second trimester was used to define anemia.Based on the severity, women with Hgb value of (10 g/dL ≤ Hgb < 11 g/dL) at first and third trimesters and (10 g/dL ≤ Hgb < 10.5 g/dL) at second trimester were classified as mild anemic. Pregnant women who had a Hgb value of (7 g/dL ≤ Hgb < 10 g/dL) and (Hgb < 7 g/dL) were categorized as moderate and severe anemic, respectively, regardless of their gestational age [45]. Manufacturer references were used to define the normal ranges for MCV (80.0–100.0 fl), MCH (27.0–33.5 pg), and MCHC (32.0–36.0 g/dL).

### 2.5. Data Processing and Analysis

Data were entered to EPI info version 3.5.3 and then transferred to SPSS version 20 statistical package for analysis. Descriptive and summary statistics were carried out using percentages and mean ± SD and were presented in tables and graphs. Binary logistic regression analysis was conducted to evaluate the difference in anemia prevalence across the relevant variables. Odds ratio, Chi-square, and 95% CI for odds ratio were computed to assess the strength of association and statistical significance in bivariate analysis. Independent variables having *P* less than or equal to 0.2 in univariate analysis were included in multivariate analysis to control confounders in regression models. Variables having *P* value less than 0.05 in multivariate binary logistic regression model were considered to be statistically significant.

### 2.6. Ethical Clearance

The study was approved by institutional review board of University of Gondar. The purpose and importance of the study were explained to each study participants. Written consent was obtained from each woman. To ensure confidentiality of participants, information, anonymous typing was used whereby the name of the participants and any participants' identifier were not written on the questionnaire, and, also during the interview to keep the privacy, they were interviewed alone. Results were communicated with clinicians working in ANC unit for appropriate management.

## 3. Result

### 3.1. Characteristics of the Study Participants

A total of 302 pregnant women with a mean (±SD) age of 26.47 ± 5.24 years were included in the study. The majority 242 (80.1%), 284 (94%), 250 (82.8%), and 194 (64.2%) were urban dwellers, married, had attended primary school and above, and house wives by occupation, respectively. The average monthly income of the participants was 1860 Ethiopian Birr (EB) and 147 (48.7%) were living with three to four family members (Table 1).

Concerning obstetrical history, 57.3% were multigravida, of whom 52.7% had an interpregnancy interval of more than or equal to 24 months and 23.7% experienced abortion. Nearly 70% of the study participants were at third trimester. Assessment of medical condition of the participant revealed that 72.5% had a normal BMI, 95.4% had no history of chronic diseases, and 4.6% had history of previous surgery. Laboratory investigation showed that 10.3% and 26.5% of the participants were reactive for HIV and infected with one or more than one intestinal parasites, respectively. *A*.* lumbricoides* (34.1%), *hookworm* (25.3%), and *E. histolytica/dispar* (17.2%) were the predominant parasites found (Table 2).

The dietary habit and nutritional assessment revealed 19.8% did not take animal products in their current pregnancy, and 42.4% had a habit of eating green vegetables on monthly and above basis. About 80.1% had a habit of drinking coffee and tea after meal (data not shown). In their current pregnancy, 44.7%, 41.4%, and 7.3% took iron sulfate, folic acid, and multivitamin tables as nutritional supplement, respectively (Table 3).

### 3.2. Prevalence and Predictors of Anemia

**Figure 1:**
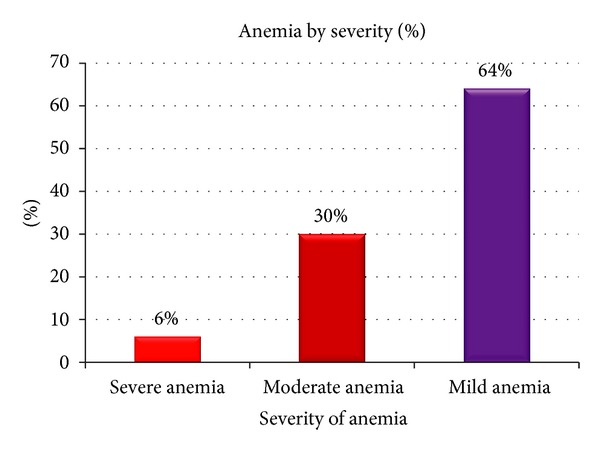
Percentage of anemia by severity among anemic pregnant women (*n* = 50).

The mean Hgb level of pregnant women was 11.96 ± 1.37 g/dL (range: 5.85–17.05 g/dL) and the overall prevalence of maternal anemia was 16.6% (*n* = 50). Of the anemic women, 6%, 30%, and 64% were severely, moderately, and mildly anemic, respectively (Figure 1).

Based on red blood cell morphologic classification of anemia, of the total anemic pregnant mothers, 76% had normocytic normochromic anemia and 14% had microcytic hypochromic type of anemia (Table 4).

High prevalence of anemia was observed in those pregnant women who were living with more than four family members (36.4%), illiterate (25.7%), and whose monthly family income < 1000EB (22%) (Table 1). In addition, high prevalence rate of anemia was found among mothers who were HIV seropositive (38.7%), infected with *hookworm* (34.8%), underweighted (30%), with more than four gravidae (32.3%), having chronic disease (27.3%), and at 3rd trimester (18.9%) (Table 2).

The prevalence of anemia among those who had a habit of eating animal products in their food stuff, not having a habit of eating vegetable, and who take tea/coffee after meal was 17.8%, 22.4%, and 15.3%, respectively. About 18.6% and 17.5% pregnant women who did not take iron sulphate and folate as nutritional therapy, respectively, were anemic (Table 3).

In bivariate analysis illiteracy, low monthly family income, large family size, underweight, gravidity*, hookworm* infection, and HIV seropositivity were significantly associated with maternal anemia. But in multivariate logistic regression analysis controlling the possible cofounders, only low monthly family income (AOR = 3.15, 95% CI: 1.19, 8.33), large family size (AOR = 4.13, 95% CI: 1.62, 10.52),* hookworm* infection (AOR = 5.75, 95% CI: 2.40, 13.69), and HIV seropositivity (AOR = 2.72, 95% CI: 1.014, 7.25) remained being independent predictors of pregnancy anemia (Table 5).

## 4. Discussion

The overall prevalence of anemia was 16.6% (95%CI (12.6, 20.6)). This prevalence was comparable to studies conducted in Trinidad and Tobago (15.3%) [16], Thailand (20.1%) [17], Zurich (18.5%) [45], Hawassa (15.3%) [39], and Gondar town (22%) [46].

The prevalence is considerably lower than previous study reports from Malaysia (35%), Jordan (34.7%), Vietnam (43.2%), Southeastern Nigeria (76.9%), Eastern Sudan (62.6%), and Jimma, Ethiopia (38.2%) [22, 23, 25, 35, 38, 41]. The possible reason for the difference may be resulted from geographical variation of factors across different areas. In addition, lower prevalence can be attributed to gradual improvement of life style and living standards and health seeking behavior by the effort of government to achieve the Millennium development goal aimed to reduce the maternal mortality by three-quarter by year 2015. In support of this argument, the prevalence of anemia in women of age 15–49 years had decreased from 27% in 2005 to 17% by the year 2011 in Ethiopia [47].

In this study, mild anemia was common followed by moderate anemia. This is consistent with reports from Africa and elsewhere in the world [23, 25, 29, 31, 39]. This study tried to demonstrate the common morphological characteristic of anemia among pregnant mothers. Of the total anemic pregnant women, 76% had normocytic normochromic anemia followed by microcytic hypochromic type which is in agreement with a report from Turkey [18] and Azezo, Gondar town [46].

This study demonstrated that mothers who have low monthly family income were three times more likely to be anemic as compared to those with high monthly family income. This is in agreement with some studies [24, 29] and contradicted to other reports [22, 23, 40, 41]. According to the 2007 Ethiopian central statistical agency household income consumption and expenditure survey, more than 57% of the total expenditure is spent on food [48]. Moreover, in this study, 80% of study participants were from urban area suggesting that they are food net buyers. As income is low, the expenditure for food becomes low. Besides, due to food price inflation, the purchasing power of income is low. So, low income groups did not get adequate nutrition and thereby low family income groups were at risk of anemia.

According to the results of our study, pregnant mothers who had been living within a family of more than four members were more likely to be anemic compared to those living with ≤ 2 family members. Nevertheless, in Jordan [23], there was no significant difference of anemia prevalence between groups of pregnant mothers living with varying family sizes. This difference may be attributed as in Jordan case; the study was undertaken in rural district where there was not great variation in family size and income. But, in this study, 80% of pregnant women were in urban areas having varying income levels and 20% in rural areas with varying family size.

This study also showed that the proportion of anemia among pregnant women who had been infected with HIV was significantly higher compared to those noninfected that is six times at higher risk. This is in line with previous studies [29, 31–33, 35]. This increased prevalence of anemia among HIV seropositive pregnant women may be explained by the fact that HIV infection is associated with lower serum folate, vitamin B12, and ferritin in pregnancy [31]. In addition, Anemia in HIV/AIDS patients may arise from a number of causes, including deregulation of the host immune system leading to destruction or inhibition of hematopoietic cells [49].

In our study, *hookworm* has increased the risk of being anemic and this finding was consistent with other studies [26, 41, 46]. This is because adult *hookworm* parasites attach and injure upper intestinal mucosa and also ingest blood. This brings about gastrointestinal blood loss and induces depletion of iron, folic acid, and vitamin B12 that ultimately anemia [13, 50].

Even though it was not statistically significant in multivariate logistic regression (but significant in bivariate analysis), multigravida and grand gravida had high odds for anemia as compared to primigravidae. Likewise, studies in Malaysia [22], Burkina Faso [29], Sudan [38], and Jimma [41] reported that gravidity did not have statistically significant contribution for difference in anemia prevalence. Despite this, a study from Trinidad and Tiago, multigravida had significantly increased likelihood of being anemic than primigravidae [16]. The disparity may be as a result of sociodemographic characteristic difference between study participants. In this study, participants who were multigravida had the following characteristics. 90% had normal and above BMI, 78% were urban residents, and 50% of them had middle and high monthly family income. These situations may reduce the risk of anemia in multigravida pregnant mothers participated in this study.

In this study, supplementation of iron sulphate, folic acid, and multivitamin during the current pregnancy period did not significantly reduce the prevalence of anemia as compared to those who did not take these supplementations. The finding was in contradiction with other studies [19–21, 25, 26, 28]. The possible reason may be that, in anemic pregnant women, these nutritional supplements were more likely to be prescribed as an intervention for management of anemia in their previous ANC visit. This needs a further study to explicitly explain how much effective the current WHO nutritional supplementation recommendation program is being implemented for prevention and control of anaemia in pregnant women [51].

### 4.1. Limitations of the Study

One of the limitation of this study is the nature of the study design its self, being as a cross-sectional study design, it does not show which preceded anemia or risk factors. Due to constraint of time and resource, stool concentration technique and parasite density were not done so we could not assess the impact of parasite load on the severity of anemia. In addition to this, the low sensitivity of wet mount to detect parasite in patient with low parasite load may underestimate the prevalence of intestinal parasite and alter odds ratio. The other limitation is that this study was conducted at tertiary care hospital located at Gondar town and majority of the study participants were urban residents. But many of the pregnant women in that district were living in rural areas where access to antenatal facilities is limited, so the prevalence of anemia would have been even more if the study was done in the general population.

## 5. Conclusion

In conclusion, the prevalence of anemia among pregnant women was high especially at third trimester. Mild type of anemia was the commonest one. Morphologically, the predominant type of anemia was normocytic normochromic, followed by microcytic hypochromic anemia. Low family income, high family size, *hookworm* infection, and living with HIV/AIDS were the main predictors of maternal anemia. To reduce the prevalence, there is a need to strengthen health care seeking behavior of women to ensure early diagnosis and management of HIV, hookworm, anemia, and other medical conditions. There is also a need to encourage family planning, and design policies and strategies pertinent to reduction of anemia in low income groups. A large community based study needs to be done to determine the prevalence and predictors of anemia in the general population of pregnant women. Besides, further studies using micronutrient assay techniques which are sensitive for the detection of latent anemia before the change of RBC morphology and indices takes place have to be conducted.

## Figures and Tables

**Table 1:** Sociodemographic characteristics of pregnant women and prevalence of anemia by sociodemographic characteristics (*n* = 302).

Variable	Anemia		Total (%)	COR (95% CI)
	Yes	No		
Age				
year	23 (15.8)	123 (84.2)	146 (48.3)	1
25–29 years	13 (15.9)	69 (84.1)	82 (27.2)	1.01 (0.48, 2.11)
≥30 years	14 (18.9)	60 (81.1)	74 (24.5)	1.25 (0.6, 2.60)
Residence				
Rural	11 (18.3)	49 (81.7)	60 (19.9)	1.17 (0.56, 2.45)
Urban	39 (16.1)	203 (83.9)	242 (80.1)	1
Marital status				
Married	47 (16.5)	237 (83.5)	284 (94)	1
Others*	3 (16.7)	15 (83.3)	18 (6)	1.01 (0.28, 3.62)
Maternal educational status				
Illiterate	18 (25.7)	52 (71.3)	70 (23.2)	3.31 (1.29, 8.54)*
Primary school	5 (9.4)	48 (90.6)	53 (17.5)	0.997 (0.30, 3.33)
Secondary school	20 (19)	85 (81)	105 (34.8)	2.25 (0.89–5.65)
Tertiary	7 (9.5)	67 (90.5)	74 (24.5)	1
Occupation				
House wife	38 (19.6)	156 (80.4)	194 (64.2)	2.27 (0.91, 5.67)
Government employed	6 (9.7)	56 (90.3)	62 (20.5)	1
Other**	6 (13)	40 (87)	46 (15.2)	1.4 (0.42, 4.65)
Family monthly income				
EB	30 (21.9)	107 (78.1)	137 (45.4)	3.22 (1.28, 8.13)*
1000–2575 EB	14 (15.6)	76 (84.4)	90 (29.8)	2.12 (0.77, 5.81)
EB	6 (8)	69 (92)	75 (24.8)	1
Family size				
≤2	16 (13.10)	106 (86.9)	122 (40.4)	
3-4	22 (15)	125 (85)	147 (48.7)	1.17 (0.58, 2.34)
≥5	12 (36.4)	21 (63.6)	33 (10.9)	3.79 (1.56, 9.17)*

*Significant (*P* < 0.05) in bivariate analysis; other* include single, divorced, and widowed; other** include private employed, farmers, merchants, and students.

**Table 2:** The prevalence of anemia according to the obstetrics and medical factors (*n* = 302).

Variable	Anemic		Total (%)	COR (95% CI)
	Yes (%)	No (%)		
Gravidity				
Primigravidae	15 (11.6)	114 (88.4)	129 (42.7)	1
Secundigravidae	11 (14.3)	66 (85.7)	77 (25.5)	1.27 (0.55, 2.92)
3-4 gravidae	14 (21.5)	51 (78.5)	65 (21.5)	2.09 (0.94, 4.65)
≥5 gravidae	10 (32.3)	21 (67.7)	31 (10.3)	3.62 (1.43, 9.17)*
History of abortion				
Yes	8 (19.5)	33 (80.5)	41 (23.7)	0.94 (0.391, 2.27)
No	27 (20.4)	105 (79.6)	132 (76.3)	1
Interpregnancy interval				
1st pregnancy	15 (11.6)	114 (88.4)	129 (42.7)	1
	2 (14.3)	12 (85.7)	14 (4.6)	1.27 (0.26, 6.21)
≥24 months	33 (20.8)	126 (79.2)	159 (52.7)	2 (1.03, 3.86)
Gestational age				
1st trimester	1 (12.5)	7 (87.5)	8 (2.6)	1
2nd	12 (12.2)	86 (87.8)	98 (32.5)	0.98 (0.11, 8.62)
3rd	37 (18.9)	159 (81.1)	196 (64.9)	1.63 (0.19, 13.65)
Body mass index				
Underweight	9 (30)	21 (70)	30 (10)	2.42 (1.03, 5.64)*
Normal and above	41 (15.1)	231 (84.9)	272 (90)	1
Presence of chronic disease				
Yes	4 (27.3)	11 (72.7)	14 (4.6)	1.4 (0.38, 5.21)
No	47 (16.3)	241 (83.7)	288 (95.4)	1
Presence of peptic ulcer disease				
Yes	20 (18.3)	89 (81.7)	109 (36.1)	1.22 (0.66, 2.27)
No	30 (15.5)	163 (84.5)	193 (63.9)	1
History of previous surgery				
Yes	2 (14.3)	12 (85.7)	14 (4.6)	0.83 (0.181, 3.84)
No	48 (16.7)	240 (83.3)	288 (95.4)	1
Malaria attack in current pregnancy				
Yes	3 (20)	12 (80)	15 (5)	1.28 (0.347, 4.7)
No	47 (16.4)	240 (83.6)	287 (95)	1
Hookworm infection				
Yes	8 (34.8)	15 (65.2)	23 (7.6)	3.01 (1.20, 7.52)*
No	42 (15.1)	237 (84.9)	279 (92.4)	1
HIV infection				
Reactive	12 (38.7)	19 (61.3)	31 (10.3)	3.87 (1.74, 8.62)*
Nonreactive	38 (14)	233 (86)	271 (89.7)	1
Syphilis				
Reactive	3 (27.3)	8 (72.7)	11 (3.6)	1.95 (0.5, 7.61)
Nonreactive	47 (16.2)	244 (83.8)	291 (96.4)	1

*Significant (*P* < 0.05) in bivariate analysis. Chronic disease comprises hypertension, kidney disease, cardiac problems, and diabetes mellitus.

**Table 3:** Prevalence of anemia in relation to dietary habit, ANC followup, and nutrient supplementation at their current pregnancy period (*n* = 302).

Variable	Anemia		Total (*N* = 302)	COR (95% CI)
	Yes	No		
Eating meat and animal products				
Yes	43 (17.8)	199 (82.2)	242 (80.1)	1
No	7 (11.7)	53 (88.3)	60 (19.9)	0.61 (0.26, 1.44)
Eating green leafy vegetables				
Yes	39 (15.4)	214 (84.6)	253 (83.8)	1.00
No	11 (22.4)	38 (77.6)	49 (16.2)	1.6 (0.75, 3.372)
Taking fruit after meal				
Yes	34 (16.6)	172 (83.5)	206 (68.2)	1
No	16 (16.7)	80 (83.3)	96 (31.8)	1.012 (0.53, 1.94)
Taking coffee or tea immediately after meal				
Yes	37 (15.3)	205 (84.7)	242 (80.1)	0.65 (0.32, 1.32)
No	13 (21.7)	47 (78.3)	60 (19.9)	1
ANC followup during current pregnancy				
Yes	28 (16.1)	146 (83.9)	174 (57.6)	1
No	22 (17.2)	106 (82.8)	128 (42.4)	1.08 (0.59, 1.99)
Iron sulphate table intake in current pregnancy				
Yes	19 (14.1)	116 (85.9)	135 (44.7)	1
No	31 (18.6)	136 (81.4)	167 (55.3)	1.39 (0.75, 2.59)
Folic acid intake in current pregnancy				
Yes	19 (15.2)	106 (84.8)	125 (41.4)	1
No	31 (17.5)	146 (82.5)	177 (58.6)	1.19 (0.64, 2.2)
Multivitamin intake in current pregnancy				
Yes	3 (13.6)	19 (86.4)	22 (7.3)	1
No	47 (16.8)	233 (83.2)	280 (92.7)	1.28 (0.36, 4.49)

**Table 4:** Distribution of morphologic type anemia among study participants.

Morphologic type of cells	Anemic status		
	Anemic	Not anemic	Total
	*n* (%)	*n* (%)	*n* (%)
Microcytic hypochromic (MCV < 80 fl, MCH < 27 pg)	8 (16%)	1 (0.4%)	9 (3.0%)
Normocytic Normochromic (MCV and MCH within the normal value)	38 (76%)	235 (93.2%)	273 (90.4%)
Macrocytic normochromic (MCV > 100 fl, MCH (27 pg < MCH < 33.5 pg))	2 (4%)	5 (1.98%)	7 (2.3%)
Other combinations	2 (4%)	11 (4.37%)	13 (4.3%)

Total	50 (16.6%)	252 (83.4%)	302 (100%)

**Table 5:** Multivariate binary logistic regression analysis of pregnancy anemia with predictor variables (*n* = 302).

Variables	Anemia		Total	OR (95% CI)	
	Yes	No		COR (95% CI)	AOR (95% CI)
Maternal educational status					
Illiterate	18 (25.7)	52 (74.3)	70	**3.31 (1.29, 8.54)**	0.61 (0.14, 2.68)
Primary	5 (9.4)	48 (90.6)	53	0.997 (0.30, 3.33)	0.37 (0.08, 1.73)
Secondary	20 (19)	85 (81)	105	2.25 (0.89–5.65)	0.99 (0.30, 3.22)
Tertiary	7 (9.5)	67 (90.5)	74	1	1
Family income/month					
Low (<1000 birr)	30 (21.9)	107 (78.1)	137	3.22 (1.28, 8.13)	3.15 (1.19, 8.33)*
Medium (1000–2575 birr)	14 (15.6)	76 (84.4)	90	2.12 (0.77, 5.81)	1.80 (0.62, 5.18)
High (>2575 birr)	6 (8)	69 (92)	75	1	1
Family size					
≤2	16 (13.1)	106 (86.9)	122	1	1
3-4 members	22 (15)	125 (85)	147	1.17 (0.58, 2.34)	1.03 (0.49, 2.13)
≥5 members	12 (36.4)	21 (63.6)	33	3.78 (1.56, 9.17)	4.13 (1.62, 10.52)**
Body mass index					
Underweight (<20 kg/m^2^)	9 (30)	21 (70)	30	**2.42 (1.03, 5.64)**	2.27 (0.83, 6.21)
Normal and above (≥20 kg/m^2^)	41 (15.1)	231 (84.9)	272	1	1
Gravidity					
Primigravidae	15 (11.6)	114 (88.4)	129	1	1
Secungravidae	11 (14.3)	66 (85.7)	77	1.27 (0.55, 2.91)	1.1 (0.37, 3.08)
3-4 gravidae	14 (21.5)	51 (78.5)	65	2.09 (0.94, 4.65)	2.19 (0.68, 6.99)
≥5 gravidae	10 (32.3)	21 (67.7)	31	**3.62 (1.43, 9.17)**	1.87 (0.31, 11.36)
Hookwarm					
Yes	8 (34.8)	15 (65.2)	23	3.01 (1.2, 7.52)	2.72 (1.01, 7.25)*
No	42 (15.1)	237 (84.9)	279	1	1
HIV					
Yes	12 (38.7)	19 (61.3)	31	3.9 (1.74, 8.62)	5.75 (2.4, 13.69)***
No	38 (14)	233 (86)	271	1	1

Bold numerical values indicate significant in bivariate but not in multivariate analysis. *Significant (*P* < 0.05), **significant (*P* < 0.01), and ***highly significant (*P* < 0.001) in multivariate analysis.
